# RecoverNow: A mobile tablet-based therapy platform for early stroke rehabilitation

**DOI:** 10.1371/journal.pone.0210725

**Published:** 2019-01-25

**Authors:** Michael Pugliese, Tim Ramsay, Rany Shamloul, Karen Mallet, Lise Zakutney, Dale Corbett, Sean Dukelow, Grant Stotts, Michel Shamy, Kumanan Wilson, Julien Guerinet, Dar Dowlatshahi

**Affiliations:** 1 School of Epidemiology and Public Health, University of Ottawa, Ontario, Canada; 2 Ottawa Hospital Research Institute, Ottawa, Ontario, Canada; 3 Champlain Regional Stroke Network, Ottawa, Ontario, Canada; 4 The Ottawa Hospital, Ottawa, Ontario, Canada; 5 Canadian Partnership for Stroke Recovery, Ottawa, Ontario, Canada; 6 Department of Cellular and Molecular Medicine, University of Ottawa, Ottawa, Ontario, Canada; 7 Brain and Mind Research Institute, University of Ottawa, Ottawa, Ontario, Canada; 8 Department of Clinical Neurosciences, University of Calgary, Calgary, Alberta, Canada; University of Birmingham, UNITED KINGDOM

## Abstract

**Introduction:**

Stroke survivors frequently experience a range of post-stroke deficits. Specialized stroke rehabilitation improves recovery, especially if it is started early post-stroke. However, resource limitations often preclude early rehabilitation. Mobile technologies may provide a platform for stroke survivors to begin recovery when they might not be able to otherwise. The study objective was to demonstrate the feasibility of RecoverNow, a tablet-based stroke recovery platform aimed at delivering speech and cognitive therapy.

**Methods:**

We recruited a convenience sample of 30 acute stroke patients to use RecoverNow for up to 3 months. Allied health professionals assigned specific applications based on standard of care assessments. Participants were encouraged to take home the RecoverNow tablets upon discharge from acute care. The study team contacted participants to return for a follow-up interview 3 months after enrollment. The primary outcome of interest was feasibility, defined using 5 facets: recruitment rate, adherence rate, retention rate, the proportion of successful follow-up interventions, and protocol deviations. We tracked barriers to tablet-based care as a secondary outcome.

**Results:**

We successfully recruited 30 of 62 eligible patients in 15 weeks (48% recruitment rate). Participants were non-adherent to tablet-based therapy inside and outside of acute care, using RecoverNow for a median of 12 minutes a day. Retention was high with 23 of 30 patients participating in follow-up interviews (77% retention rate) and all but 3 of the 23 interviews (87%) were successfully completed. Only 2 major protocol deviations occurred: one enrollment failure and one therapy protocol violation. Barriers to tablet-based care were frequently encountered by study participants with many expressing the assigned applications were either too easy or too difficult.

**Conclusions:**

Acute stroke patients are interested in attempting tablet-based stroke rehabilitation and are easily recruited early post-stroke. However, tablet-based therapy may be challenging due to patient, device and system-related barriers. Reducing the frequency of common barriers will be essential to keeping patients engaged in tablet-based therapy.

## Introduction

Stroke survivors frequently experience one or more of a broad range of deficits (eg. aphasia, weakness, sensory loss, cognitive impairment) requiring post-stroke rehabilitation. Globally, it is estimated that around one-third of survivors experience communication deficits [1], up to two-thirds experience cognitive deficits [2], another two-thirds experience upper limb impairments [3], and around one-third experience post-stroke depression [4]. Stroke rehabilitation improves these deficits with more intense therapy and earlier therapy initiation being associated with better recovery [5–9]. However, an optimal window for initiating rehabilitative therapies has not been established [7]. Although current guidelines recommend that stroke survivors begin rehabilitation within 5–7 days post-stroke [10], this goal is not met in many jurisdictions due to a lack of therapists with expertise in stroke recovery and challenges in accessing rehabilitation facilities [10–13]. While waiting in acute care to begin rehabilitation, patients spend little to no time engaging in activities to promote recovery [11].

The ubiquity of smartphones, mobile application (apps), and mobile-tablet computers has brought along with it an interest in leveraging this technology for the purposes of providing stroke rehabilitation. There have been numerous studies focusing on mobile tablets as rehabilitation platforms for a variety of post-stroke deficits including communication [14–22], cognition [16,23,24], fine-motor skills [25–28]. Stroke survivors have typically expressed high satisfaction with tablet-based stroke therapies [14,15,18,20,24,25,27–29] and have pointed to therapy independence [18,25,29] and the convenience of being able to engage in therapy at home [15,18] as specific positive aspects. However, most research has focused on patients in the chronic phases of stroke [30,31], long after their stroke incident occurred and where therapy is associated with the least amount of recovery [7]. Few studies of tablet-based stroke therapy have focused on survivors in acute care during the early phases of stroke recovery where there is an opportunity for patients to engage in rehabilitative therapies, and during a time when therapy is associated with the greatest recovery.

Currently-available mobile technologies may provide an opportunity to engage in rehabilitation in acute care while patients wait to begin traditional therapist-led stroke rehabilitation, and to enhance concurrent traditional rehabilitation outside of acute care during the early stages of stroke recovery. To the best of our knowledge, our previous studies in this area are the only to have offered tablet-based stroke therapy to stroke survivors in acute care shortly after their stroke [30,31]. We have demonstrated the feasibility of implementing RecoverNow, a mobile tablet-based therapy platform for post-stroke communication deficits in the acute setting [20]. A patient engagement survey followed and showed patient interest in beginning tablet-based stroke rehabilitation within days of stroke and in continuing tablet-based therapy after discharge from acute care [24]. A period of development followed these studies expanding RecoverNow into a more comprehensive rehabilitation platform including cognitive and fine-motor therapy (often the therapeutic domain of occupational therapists) in addition to speech language therapy. A self-report depression screen was also included to explore the feasibility of administering stroke care guideline recommended depression screening using a mobile tablet. The platform was re-designed to include security measures that allowed patients to take RecoverNow outside of acute care, and with a therapist-only web-based administration portal with tablet usage monitoring features that allowed therapists to track patient therapy engagement and to remotely modify therapy content. The purpose of this study was to establish the feasibility of the new iteration of RecoverNow designed for acute stroke patients with speech language therapy (SLT) and/or occupational therapy (OT) needs across the continuum of care from the acute setting to discharge destination.

## Methods

### Sampling

An unblinded, single-group, prospective cohort design was used, and a convenience sample of 30 patients was recruited from the neurology ward of the Ottawa Hospital Civic Campus in Ottawa, Ontario, Canada by either a speech language pathologist or occupational therapist.

### Eligibility criteria

We approached patients to participate if (1) they were admitted under neurology with a confirmed diagnosis of stroke, and (2) presented with mild to moderate communication and/or cognition deficits based on speech language pathologist or occupational therapist judgement and/or mood symptoms and/or patients with score ≥ 1 on the National Institute of Health Stroke Score (NIHSS). We excluded patients with (1) pre-stroke speech, language disorders and/or cognitive disorders as we were interested only in the feasibility of the device acute, stroke-induced deficits (2) pre-existing severe disability from any cause that, in the opinion of the investigator, rendered the patients unable to complete the tasks required by the study (ex: moderate to severe dementia, functionally dependent for activities of daily living), or (3) with severe comprehension deficits that would prevent participants from being able to understand study-related instructions.

### Procedure

Participants were given a RecoverNow tablet to use for three months and were invited by phone to participate in a follow-up interview at the end of this period. Patient characteristics were collected at baseline, tablet usage was collected across the three month period, and health outcome data was collected at the three month follow-up interview.

### Intervention

A speech language pathologist or occupational therapist prescribed participants preselected, modality-specific therapeutic apps for stroke-induced deficits related to communication, cognition, and fine-motor ability based on individualized standard of care assessments (S1 Table). Selected apps were either designed specifically with therapy in-mind (apps like Constant Therapy, and Dexteria) or were thought to be analogous to traditional face-to-face therapy exercises (apps with memory games or that involved repetitive goal-oriented movement). Once apps had been selected, therapists signed into RecoverNow’s web-based administration portal where they could view participant therapy schedules and assign a personalized daily therapy schedule consisting of specific apps and recommended usage times for each app. Once assigned, these apps became available on that participant’s RecoverNow tablet. The number of apps assigned to each participant varied depending on their clinical profile but the recommended daily therapy time totaled 1 hour regardless of the number of apps assigned. Therapists could return to administrative portal using any internet connected device to view participant app usage statistics. Participants were given a brief training session by either an allied health professional or research staff member familiar with the RecoverNow platform.

This iteration of RecoverNow also included a depression screen in the form of a tablet-integrated Patient Health Questionnaire (PHQ9). We were interested in the feasibility of tablet-based depression screening as severe depression is known to interfere with rehabilitation, and should treated first before engaging in rehabilitative therapies. Patients were instructed on how to complete the depression screening during training and were encouraged to complete the PHQ9 independently if possible and if not, a member of the research team assisted. If a patient screened positive for depression on the PHQ9, the investigator directly alerted the clinical team; formal assessment and/or treatment was at the discretion of the clinical team. Participants could take the RecoverNow tablets with them upon discharge from acute care for up to three months post-enrollment with the understanding that they would attempt to engage in tablet-based therapy using RecoverNow for at least one hour a day. Participants were trained to use RecoverNow’s messaging feature that could be used to notify therapists if they were displeased with the content of their assigned therapy. Therapists could view these messages in the administrative portal and modify the assigned apps.

### Monitoring of participant usage after discharge

Every effort was made to follow-up with participants one day after assigning a tablet, or after extended periods (three or more days) of non-usage as tracked by the webpage administration portal. Follow-up was done either in-person or via phone to identify reasons for lack of therapy adherence. If a participant continually demonstrated a lack of usage and did not express a desire to continue using the RecoverNow tablet, a decision to end their involvement with the study was made by the primary investigator. Once a participant reached the study endpoint, arrangements were made by research personnel to retrieve the tablet.

### Primary outcome: Feasibility

Five criteria were used to establish feasibility and inform a future randomized controlled trials (RCT) of treatment efficacy (Table 1).

**Table 1:** The five facets of mobile tablet-based therapy feasibility.

Facet	Definition	Justification
Recruitment rate	The number of patients enrolled divided by the total number admitted with stroke until the study sample was met.	Will be used to determine the total sample size and number of RCT sites.
Adherence rate	The number of patients who completed the full course of the intervention divided by the total number enrolled.	Will be used to inform therapy tolerability.
Retention rate	The number of patients presenting for the 12-week follow-up assessment divided by the total number enrolled.	Will be used to adjust the final RCT sample size calculation.
The proportion of successful follow-up interviews	The number of patients who successfully completed the interview divided by the total number of interview participants.	Will be used to determine the acceptability of the follow-up interview and to predict attrition rates.
Protocol deviations	Deviation related to inclusion/exclusion criteria violations and deviations from therapy protocols.	Will be used to assist with the fine tuning of the RCT protocol.

#### Follow-up interview

All participants, regardless of therapy completion and adherence, were invited to participate in an in-person follow-up interview three months after their initial enrollment. Participants were asked to be interviewed within one week of their scheduled three-month completion date, and were invited to conduct a phone interview if unable or unwilling to travel to the Ottawa Hospital for an in-person interview. The final in-person interview consisted of the National Institutes of Health Stroke Scale (NIHSS) [32], Barthel Index (BI) [33,34], Modified Rankin Scale (mRS) [35], and Patient Health Questionnaire (PHQ9) [36–38]. These measures were selected based on their strengths as outcome measures for and frequency of use in clinical trials. The phone-interview consisted of these same measures except the NIHSS. The purpose of the follow-up interview and the measures selected related strictly to providing an estimate for the proportion of successful interviews for future clinical trials; not to demonstrate efficacy or health improvements over time. Therefore, no effort was made to take baseline measurements with the exception of the PHQ9 which was administered as part of investigating the feasibility of tablet-based depression screening.

### Secondary outcome: Barriers to care

We tracked barriers to mobile tablet-based care as a secondary outcome. These barriers were identified to help further refine the RecoverNow platform, improve the protocol of a future randomized controlled trial, and better integrate the platform into current standard stroke care. We organized barriers to tablet-based care into device-, patient-, or system-specific barriers. Although a low risk therapy, we tracked adverse events even if unrelated to the intervention.

### Data analysis

The analysis used descriptive statistics to summarize the characteristics of the stroke participants included in the study and the therapy initiation process. A description of intervention feasibility used the five criteria discussed previously with one notable exception; adherence was lower than expected and require a more in-depth analysis than reporting a single adherence rate. The analysis of tablet usage habits explored the number of days patients could have potentially used the tablet (“potential tablet days”), the number of days the tablet was actually used by the patients (“days used”), and average daily usage in minutes. We defined one or more minute of usage throughout a day as a day used. We performed exploratory sub-group analyses to uncover any variation in usage data by therapy needs and setting. Although the study was not powered to make generalizable conclusions about sub-group differences, we carried out these analyses as a hypothesis-generating exercise. We created a descriptive summary of barriers to care and adverse events encountered during the follow-up period. Any barriers or adverse events communicated by participants to the study staff were noted in the study database and later organized into descriptive categories to provide a quantitative, descriptive summary [20,30].

Analyses of the primary and secondary outcomes included all recruited participants. A Wilcoxon sign-ranked test evaluated the significance between baseline and following PHQ9 scores. All statistical tests used a two-sided alpha set to 5% to determine statistical significance. Statistical analyses used SAS version 9.4, Microsoft Excel 2013, and IBM SPSS version 24.

### Ethical considerations

Due to the low risk of the intervention, and the difficulty stroke survivors may face providing written or verbal consent due to the nature of their deficits, a waiver of consent was obtained for this study. Standard care procedures were not interrupted and patient privacy was protected. All confidential patient information was entered into a password protected database maintained on a secure hospital server. Only study personnel had access to this document. All information contained in the tablets, administration portal and tablet-to-administration portal transmissions were encrypted. This study received Ottawa Hospital ethics approval (20140609-01H).

## Results

### Recruitment rate and participants

**Fig 1:**
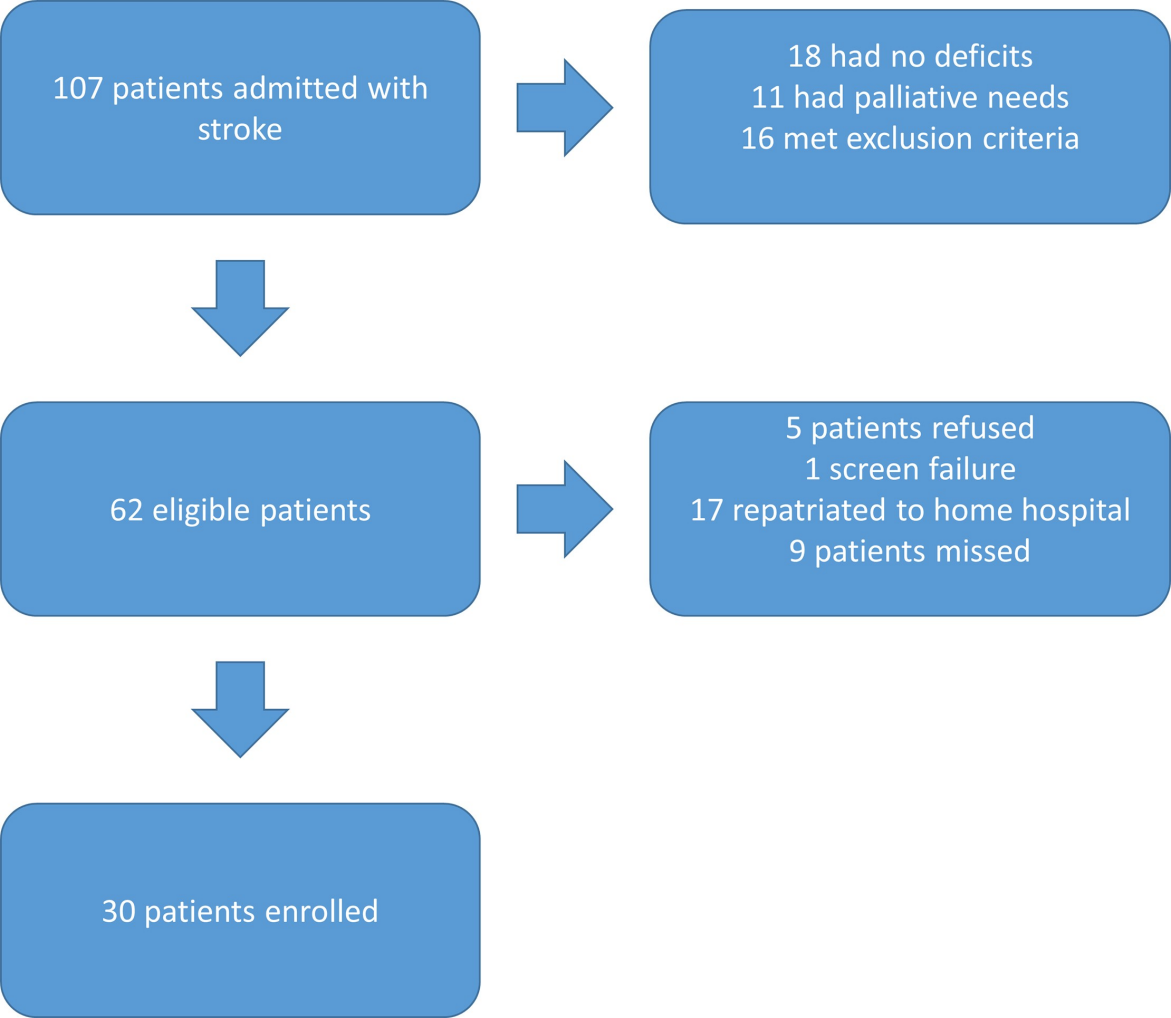
Participant flow chart.

Over 15 weeks of enrollment, 107 patients were admitted to the neurovascular unit with acute stroke (Fig 1). Of these, 62 (58%) met the inclusion criteria. Of the eligible participants, five refused to participate, one participant was too disabled to complete therapy initiation because of an inclusion/exclusion criteria screening failure, 17 were repatriated to home hospital prior to being offered the study, and nine were missed. The remaining 30 were enrolled, for a recruitment rate of 48%. Most enrolled participants (70%) had either OT or both SLT and OT needs, and over half had experience using touchscreen devices (Table 2). Although most of the strokes were ischemic and occurred in the territory of the middle cerebral artery, six patients with intracranial hemorrhage (ICH) were also recruited.

**Table 2:** Participant characteristics.

Characteristics (n = 30)	Median (Range) / n (%)
Sex (% male)	21 (70%)
Age	75 (40–95)
Type of stroke	
Ischemic	24 (80%)
Intracranial hemorrhage	6 (20%)
Education	
High school (no diploma)	6 (20%)
High school graduate	3 (10%)
College graduate	4 (13%)
University graduate	7 (23%)
Masters	7 (23%)
PhD	0 (0%)
Other	3 (10%)
Computer knowledge	
None	5 (17%)
Beginner	9 (30%)
Average	10 (33%)
Advanced	6 (20%)
Previous touchscreen device experience	18 (60%)
Therapy needs^a^	
SLT needs only	9 (30%)
OT needs only	14 (47%)
SLT and OT needs	7 (23%)
Alpha-FIM	72 (31–116)

^a^Needs were determined based on speech language pathologist or occupational therapist assessment of communication/fine-motor/cognitive deficits.

### Therapy initiation

Participants began RecoverNow within a median of four days post-stroke with the median time of admission to assessment taking 2 days, and the median time from assessment to enrollment taking 2.5 days (Table 3). RecoverNow set-up, including app assignment, training, depression screening, case-report form completion, and travel time to and from the neurovascular ward took a median time of 41 minutes. Seventy-three percent of participants were successfully screened for depression using the PHQ9, among whom half were able to complete the questionnaire independently with minimal assistance. Thirty-seven percent of screened participants screened positive for depression (score of five or above).

**Table 3:** Mobile tablet-based therapy initiation.

Therapy Initiation (n = 30)	Median (Range) / n (%)
Days post-stroke	4 (1–19)
Days post-admission	4 (1–19)
Initiated within 7-days post-stroke	21 (70%)
Set-up time (minutes)	41 (6–99)
SLT needs only	31 (15–60)
OT needs only	50 (20–94)
SLT and OT needs	35 (6–99)
Successful PHQ9 screen	22 (73%)
Independent	11 (50%)
Assisted	11 (50%)
PHQ9 results (n = 19)^a^	
Minimal depression	12 (63%)
Mild depression	6 (32%)
Moderate depression	1 (5%)
Moderate-severe depression	0 (0%)
Severe depression	0 (0%)

^**a**^Three of the 22 PHQ9 scores were lost because of a programming error in the RecoverNow administration portal.

### Tablet usage habits

Only three participants made it to the final week of the three-month follow-up with their RecoverNow tablet, all other participants having either dropped out and returned the tablet or kept the tablet but abandoned their therapy. During the course of the study, research staff identified a programming error in the collection of tablet usage data. For 13 patients, we observed random repetitions of usage statistics from particular days. Although we could not be certain that the first usage value observed was true based on the program used to collect RecoverNow usage data, there was no way to determine the true usage statistics for the days filled with repeated values. Therefore, we kept the first usage value and marked the repeated usage values as zero to prevent overestimations of tablet usage.

Participants accrued a median of 11 potential tablet days before abandoning therapy and used the device a median of five days during this time (Table 4). Overall, participants used the device half of the days they could have been engaging in therapy. Twenty-one (70%) participants agreed to take the tablet with them upon being discharge and accrued a median of 14 potential tablet days post-discharge before abandoning therapy. Post-discharge median daily usage and percentage of days used were lower compared to acute care and four participants did not use the device at all once discharged.

**Table 4:** Participant tablet usage habits overall and stratified by setting.

	Median (Range)		
	Overall	Acute Care	Post-Discharge
Participants (percent)	30 (100%)	30 (100%)	21 (70%)
Potential tablet days	11 (2–84)	4 (1–15)	14 (0–78)
Days used (≥ 1 minute)	5 (1–57)	2 (1–7)	5 (0–51)
% days used (≥ 1 minute)	50 (15–100)	60 (15–100)	38 (0–100)
Adherent days (≥ 1 hour)	0 (0–48)	0 (0–6)	0 (0–42)
Average daily usage (minutes)	12 (0–212)	13 (3–137)	10 (0–223)

#### Tablet usage habits stratified by rehabilitation needs and therapy setting

The hypothesis generating exploration of usage data showed variation in tablet usage habits between participants with different rehabilitative needs. However, it is not clear if these were true differences or products of random variation. Overall, participants with SLT needs, and both SLT and OT needs accrued the most potential tablet days before abandoning therapy, although all three groups seemed to use the device a similar number of times during this period and accrued very few adherent days. However, participants with both needs had higher median daily usage than the other two groups (Table 5). Participants with SLT needs tended to use the device every day in acute care, more than the other two participant sub-groups. All participants with only SLT needs agreed to take the device with them upon discharge compared to 50% of participants with only OT needs took the device and 71% of patients with both needs took the device.

**Table 5:** Tablet usage habits stratified by patient rehabilitation needs and therapy setting.

	Median (Range) / n (%)		
Overall	SLT needs only	OT needs only	SLT and OT needs
Participants (percent)	9 (30%)	14 (47%)	7 (23%)
Potential tablet days	16 (2–60)	8 (3–83)	17 (2–84)
Days used (≥ 1 minute)	6 (1–29)	4 (1–14)	5 (1–57)
% days used (≥ 1 minute)	45 (25–83)	51 (15–100)	50 (18–78)
Adherent days (≥ 1 hour)	0 (0–6)	0 (0–2)	1 (0–48)
Average daily usage (minutes)	11 (5–27)	9 (2–30)	22 (5–212)
Acute care			
Participants (percent)	9 (30%)	14 (47%)	7 (23%)
Potential tablet days	2 (1–8)	6 (1–15)	7 (1–9)
Days used (≥ 1 minute)	1 (1–4)	3 (1–7)	1 (1–6)
% days used (≥ 1 minute)	100 (38–100)	59 (15–100)	67 (33–100)
Adherent days (≥ 1 hour)	0 (0–1)	0 (0–1)	0 (0–6)
Average daily usage (minutes)	10 (3–33)	12 (3–43)	15 (3–137)
Post-discharge			
Participants (percent)	9 (43%)	7 (33%)	5 (24%)
Potential tablet days	14 (0–58)	14 (0–78)	64 (6–77)
Days used (≥ 1 minute)	5 (0–28)	3 (0–12)	36 (0–51)
% days used (≥ 1 minute)	38 (0–81)	15 (0–100)	58 (0–80)
Adherent days (≥ 1 hour)	0 (0–5)	0 (0–1)	12 (0–42)
Average daily usage (minutes)	10 (0–26)	2 (0–28)	23 (0–223)

### Retention rate and follow-up interviews

Follow-up interview participation was 77% with only seven participants declining both an in-person or telephone-based interview (Table 6). Only 21% of interviews were conducted during a face-to-face appointment at The Ottawa Hospital Civic Campus, and the remaining interviews were telephone-based. The most common reason for declining in-person interviews was the difficulty of commuting to the hospital. All but three (87%) of the conducted interviews were successfully completed. Two phone interviews were conducted with caregivers who could not complete the PHQ9 on the participant’s behalf. One further participant could not complete the PHQ9 because of a language barrier. Among the twelve participants who completed depression screening at both baseline and follow-up, there was no significant change in PHQ9 scores (p = 0.37).

**Table 6:** Retention rate and results of 3-month follow-up interviews.

Follow-up Interview (n = 30)	Median (Range) / n (%)
Retained for follow-up interview	23 (77%)
Interview format (n = 23)	
Face-to-face at hospital	5 (21%)
Telephone	18 (78%)
Within one week of 3-month follow-up date (n = 23)	19 (83%)
Interviews completed (n = 23)	20 (87%)
Interview results	
NIHSS (n = 5)	1 (0–5)
Modified Rankin Scale (n = 23)	2 (0–5)
Barthel Index (n = 23)	95 (5–100)
PHQ9 Score (n = 20)	3 (0–14)
PHQ9 Results (n = 20)	
Minimal depression	15 (79%)
Mild depression	3 (16%)
Moderate depression	1 (5%)
Moderate-severe depression	0 (0%)
Severe depression	0 (0%)

### Protocol deviations

There was a single deviation from inclusion/exclusion criteria where a patient screened as eligible for enrollment was too disabled to complete therapy initiation due to cognitive deficits. (Table 7). This participant was not included in the analyses presented here, nor were they part of the 30 patient convenience sample as they did not complete therapy initiation. There was a single therapy protocol deviation where a patient left before an ordered occupational therapy assessment could be performed. This participant still accrued tablet days with apps assigned for SLT needs and was thus included in the study.

**Table 7:** Summary of results for the five feasibility facets.

Facets	Median (Range) / % (n)
Recruitment rate	30/62 (48%)
Tablet usage habits (average daily usage in minutes)	12 (0–212)
Acute care (n = 30)	13 (3–137)
Post-discharge (n = 21)	10 (0–223)
Retention rate (n = 30)	23 (77%)
Completed interviews (n = 23)	20 (87%)
Protocol deviations	2

### Barriers to care

Barriers related to the therapy device, patient characteristics, and the surrounding environment or system were identified (Table 8). Participants frequently encountered barriers necessitating the need for therapists to make adjustments to the assigned applications and/or suggested time devoted to each application via the administration portal. Therapy adjustments could be made from any Internet connected device by signing-in to RecoverNow’s administrative portal and editing the applications made available on a particular participant’s device. This process typically only took a few minutes. Despite being part of the training session, only a single participant used the messaging system to communicate their discontent with the assigned therapy, likely due to the number of steps required to send a message (this was a four step process). Rather, therapists were usually only made aware of participant discontent with therapy when research staff contacted participants by phone after three or more days of non-usage.

**Table 8:** Identified barriers to mobile tablet-based stroke rehabilitation.

Device Barriers	Proposed Solution
App difficulty (too easy/too hard)	Baseline and ongoing skill-based app assignment and adjustment
Apps with poor touch responsiveness	Adjust sensitivity settings
Disliked app content	Collaborative app selection
In-app advertisements	Purchase add-free versions of apps
Language barrier	Select apps with language packs
Programming errors	Continue development
**Patient Barriers**	
Could not read	Adjust therapy, caregiver assistance
Difficulty focusing on/looking at device	None
Difficulty following instructions	Caregiver assistance
Fine-motor difficulty (dexterity/nails)	Provide stylus pen
Forgot training	Provide additional training, train caregiver
Left on trip without tablet	None
Lost charger, battery died	Case with charger slot/attachment
Too busy	None
Too tired	None
Placed out of reach	Bedside tablet sling
**System Barriers**	
Difficulty contacting patients	
Discharge to inpatient rehabilitation	Coordinate with rehabilitation centres
Discharged home (out of date information)	Collect current preferred contact information
Patients discharged without being seen	Communication with hospital staff

**Fig 2:**
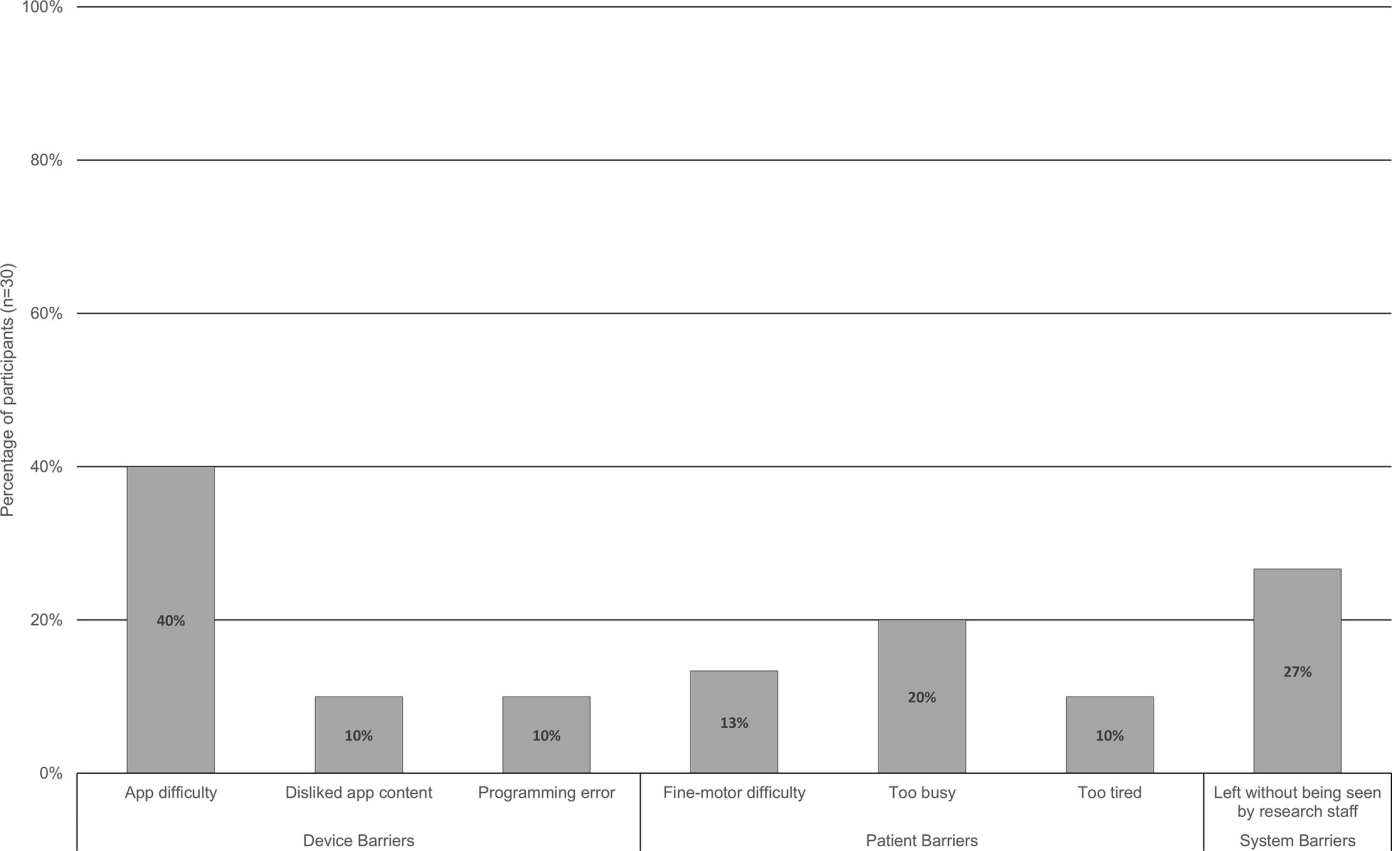
Frequently encountered barriers to tablet-based rehabilitation among acute stroke patients.

Twelve participants noted the assigned apps were either too difficult or too easy and three reported disliking app content (Fig 2). Three participants reported programming errors though these issues were not observed by research staff. Four participants had difficulty manipulating the touchscreen due to either the finger dexterity required or because of long nails. Patients faced barriers reflected of ill cognitive health; three reported being too tired to use the device, three reported difficulty focusing on or looking at the device for lengths of time, one forgot tablet training and another could no longer read. Other patient barriers were generally reflective of good patient health; six reported being too busy with their daily affairs and one participant left their tablet when they went on a trip. Eight participants were discharged without being seen by research staff, leading to multiple chargers and a tablet being lost, and another participant not being informed that they could take their tablet with them upon discharge. Contacting patients after periods on non-usage was often difficult because the participant either had outdated contact information, or were moved to an external rehabilitation center room without a telephone.

Eight participants did not complete depression screening. Most notably two participants could not complete screening due to aphasia, and another two because of a language barrier. Of the twenty-two participants completed PHQ9 screening, half could not complete screening independently. All eleven of these participants had difficulty reading or understanding the PHQ9 questions and four participants could not select response radio buttons because of their small size.

### Adverse events

Six participants experienced adverse health events unrelated to the tablet-based therapy. Three experienced further stroke events, two became terminally ill, and another developed a gastro-intestinal illness during their inpatient rehabilitation stay. These events either occurred after therapy had been abandoned or led to therapy being abandoned. One highly adherent participant was readmitted after another stroke and presented with significantly more SLT needs and new OT needs. Despite this, he continued to be highly adherent until further strokes rendered him unable to use the tablet.

## Discussion

This study determined that while RecoverNow use was feasible, few patients completed the target rehabilitation time. The recruitment of 30 patients was completed in 15 weeks with few patients being missed or declining to participate. Despite initial interest in using tablet technology for therapeutic purposes, therapy adherence was low, likely due to the barriers to care identified throughout follow-up. Most participants were retained for a follow-up interview and were able to complete an in-person or telephone interview. The study implications and limitations are discussed below.

### Recruitment rate

The ease and speed with which patients were recruited reflected the general interest by patients in using tablet technology for therapy [15,24]. During the 15 weeks of study recruitment there were 107 stroke, of which 67 (58%) met study inclusion criteria. Unexpectedly, 17 eligible patients were repatriated to their home hospital before being offered to join the study. Repatriation has become common practice for patient undergoing thrombectomy with patients being sent to The Ottawa Hospital for the procedure and then returned to their home hospital upon procedure completion. Trials will need to account for this occurrence as thrombectomy is a standard of care procedure and repatriation from procedure-performing centres is common across North America. Excluding these participants would lead to a lower recruitment rate, while partnering with nearby community hospitals would help to capture these patients and improve the number of stroke survivors eligible for participation.

### Therapy initiation

Therapy was initiated a median of four days post-stroke, earlier than previous RecoverNow studies in response to feedback from the RecoverNow patient engagement survey requesting earlier therapy [20,24], and earlier than other studies reported in the literature [30,31]. The time required to train participants was fairly short, rarely exceeding more than 1 hour. Higher functioning patients, especially those with previous tablet experience may not require as extensive training as other patients and could be left to explore the device independently. This independent discovery of the device may encourage participants to continue using the device and would lower the amount of time acute care personnel would have to dedicate to training sessions.

### Tablet-based depression screening

Tablet-based screening of stroke patients for depression appears to be feasible in the acute setting with the majority of participants successfully completing screening despite encountered barriers. The PHQ9’s presentation should be tailored to the stroke population to increase the proportion of patients who can complete screening with minimal assistance. Specifically, providing large buttons for indicated responses and large text for easy reading. However, it is likely that patients with visual neglect and certain degrees of receptive aphasia will always require assistance to complete tablet-based depression screening.

Among all participants who completed the screen, 37% of participants were positive for depression which agrees with previous post-stroke depression literature [4]. Despite depression being a common post-stroke complication and depression screening being a part of clinical guidelines, screening is often not performed [39]. Mobile tablets may provide an opportunity to improve adherence to depression screening guidelines.

### Tablet usage habits and barriers to care

We had hypothesized that patients could use their time in acute care to engage in tablet-based therapy due to the lack of activities available for promoting recovery [11]. Our initial RecoverNow study found patients with mild aphasia engaged in therapy for over two hours a day during their acute stay [20], much higher than other studies involving chronic stroke patients in other settings [14,16,18]. The next phase confirmed patient interest in using the device and that one hour a day of therapy seemed reasonable to them [24]. However, participants in this study engaged in tablet therapy far less than expected, likely due to the frequency with which they encountered barriers to tablet-based care.

Despite apps being initially prescribed based on standard of care assessment and then adjusted based on participant feedback on an ongoing basis by an experienced speech language pathologist and/or occupational therapist, participants had issues with therapy difficulty level and the fine-motor and cognitive skills required to interact with the device. These observations agree with barriers observed with chronic stroke patients [14,18,25,27,29]. Baseline and ongoing application performance-based therapy assignment could help improve patient engagement with tablet-based therapy.

The convenience and usability of tablet-based therapies have been reported as important aspects of therapy administration by participants in studies conducted by other research teams [15,18]. Given the low usage seen in this study in comparison to previous RecoverNow cohorts, it is reasonable to speculate that design flaws unique to this new iteration of RecoverNow may have been detrimental to the platform’s convenience and ease of use. Participants also reported programming errors, although these often disappeared when research staff was present. Free versions of apps were used to save costs, many of which contained ads which were difficult to close, which led to participant frustration and likely discouraged tablet use.

#### The importance of regular patient contact

Regular patient contact during the follow-up period turned out to be an important part of the therapy procedure. Only a single participant used RecoverNow’s therapist messaging system to express their displeasure with the assigned therapy. Participants may have forgotten about the messaging system or found it too complex to use as multiple steps needed to be followed. Other tablet-based therapy studies with stroke patients have used teleconferences to stay in touch with patients [23,29], although this requires a high-quality internet connection that may not be available to all stroke survivors [20,23,29]. Other studies have opted to use face-to-face meetings [19] or group therapy sessions instead of relying on technology [17]. Regardless of the method used to stay in regular contact with patients, it should be simple and convenient.

#### Patient characteristics, barriers to care, and tablet usage habits

This study offered tablet-based therapy to acute stroke patients with a broader range and severity of deficits than previous RecoverNow studies to improve our understanding of treatment feasibility. Participants with only SLT needs kept the tablet for fewer days than those with OT or both needs, tended to be less disabled, and were quickly discharged. Some patients with only SLT needs the assigned tasks too easy which likely contribute to low usage, similar to observations reported in tablet-based therapy studies involving chronic stroke patients[14,18]. The usage habits of participants with OT needs were similar to those with SLT needs, however far more of these patients declined to take the device upon discharge. Participants with both SLT and OT needs used the device the most overall perhaps due to the wider variety of apps assigned to the participants.

### Retention rate and interview completion

Participants expressed a preference for phone interviews rather than in-person interviews. Participants may not have felt required to return because they did not need to sign a consent form laying out study expectations or may not have been interested in coming for an interview that was not linked to a routine medical follow-up. Participants often noted the need to drive far distances, arrange transportation, and finding parking as deterrents. Other participants also noted significant mobility difficulties as complicating the logistics of arranging an in-person interview at the hospital and expressed a desire to perform a phone interview instead. However, most interviews were completed once initiated.

### Protocol deviations

Only two major protocol deviations related to inclusion/exclusion criteria and therapy procedures were encountered throughout the study. The clear criteria set for eligible patients and the smooth integration of a simple intervention into existing therapy procedures in the acute setting likely contributed to the small number of protocol deviations observed.

### Limitations

The focus on feasibility and characteristics of the current RecoverNow platform led to some study limitations. Patient performance on assigned applications was not measured meaning it was not possible to determine if participants were improving on therapy activities. This was a necessary trade-off, which gave therapists the flexibility to use the web-based administration portal to remotely modify the apps available on the RecoverNow tablets as participant needs changed regardless of whether they were in acute care or had been discharged. There were programming errors in the usage data causing seemingly random repetitions of certain data points. Although we were unable to recreate this error and correct it as its source, we dealt with this issue during analysis by conservatively by assigned a usage of zero minutes for the repeated values. However, we do recognize that this may have caused an underestimation of therapy usage for affected participant tablets. The usage data does not reflect whether participants were actively engaging with the applications or idly interacting with the device. However, a threshold was set to try and separate non-significant tablet activity from true attempts at engaging in therapy. Although there were no means of identifying who actually used the RecoverNow tablets, therapists and research staff made it clear during the training phase that the tablet was solely intended to be used for the patient’s recovery activities.

## Conclusions

Acute stroke patients are interested in attempting mobile tablet-based stroke rehabilitation and are easily recruited into tablet-based therapy studies early post-stroke. However, consistently engaging in tablet-based therapy from acute care to discharge destination may be challenging due to barriers to care. The reported findings can be used to help guide others interested in developing and providing tablet-based stroke therapy interventions. A few key points in particular are worth reiterating:

- Acute stroke patients are generally interested in attempting tablet-based therapy and are willing to start within their first-week post-stroke.
- Tablet-based depression screening appears to be feasible in the acute setting although some stroke survivors may require assistance depending on their deficits.
- The tablet-based therapy platform and its therapeutic content must be carefully matched to stroke survivor deficits to minimize barriers to care and maximize therapy compliance.
- Regular contact with patients using a simple and convenient method is important to promote consistent therapy engagement by addressing barriers to care.

## Supporting information

S1 Table RecoverNow application list. (DOCX)
