# Supplementary material for: RecoverNow: A mobile tablet-based therapy platform for early stroke rehabilitation
Source: PLoS One. 2019 Jan 25;14(1):e0210725. doi: 10.1371/journal.pone.0210725 (PMC6347149; doi:10.1371/journal.pone.0210725)
Supplement: S1 Table — (DOCX) [file pone.0210725.s001.docx]

S1 Table. RecoverNow application list.

| **Application Name** | |
| --- | --- |
| Anagram Twist | Magic Piano |
| Awesome Memory | Math Academy |
| Bingo | Memory Matches |
| Blankety-Blank | Memory Matches 2 |
| Boggle for Tablet | Morphos |
| Calculator Free | My Mosaic |
| Candy Crush | Parking Mania Free |
| Chain of Thought | Piano Free With Songs |
| Constant Therapy | Pop Words |
| Counting Money | RhymieStymie |
| Crossy Road | Scrabble |
| Dexteria | Search 4 It |
| Dr. Driving | Series 1 |
| Einstein Brain trainer HD | Slide Me Out |
| Fit Brains | Smash Hit |
| Flow Free | Solitaire |
| Fruit Ninja | Sudoku by Ticbits |
| Get+Together | Tactus Therapy |
| Glow Puzzle | This is to That |
| iVolution | Word Explorer |
| Jigty Jigsaw Puzzle | Yahtzee |
| Just Saying |  |
| Lumosity |  |
